# TCA cycle impairment leads to PIN2 internalization and degradation via reduced MAB4 level and ARA6 components in Arabidopsis roots

**DOI:** 10.3389/fpls.2024.1462235

**Published:** 2024-12-16

**Authors:** Xiaomin Song, Iwai Ohbayashi, Song Sun, Qiuli Wang, Yi Yang, Mengyuan Lu, Yuanyuan Liu, Shinichiro Sawa, Masahiko Furutani

**Affiliations:** ^1^ College of Life Sciences, Fujian Agriculture and Forestry University, Fujian, China; ^2^ Haixia Institute of Science and Technology, Fujian Agriculture and Forestry University, Fujian, China; ^3^ Department of Life Sciences, National Cheng Kung University, Tainan, Taiwan; ^4^ Haixia Institute of Science and Technology, State Key Laboratory of Ecological Pest Control for Fujian and Taiwan Crops, Fujian Agriculture and Forestry University, Fujian, China; ^5^ Institute of Industrial Nanomaterial (IINA), Kumamoto University, Kumamoto, Japan; ^6^ International Research Organization for Advanced Science and Technology (IROAST), Kumamoto University, Kumamoto, Japan; ^7^ International Research Center for Agricultural and Environmental Biology (IRCAEB), Kumamoto University, Kumamoto, Japan; ^8^ Graduate School of Sciences and Technology, Kumamoto University, Kumamoto, Japan; ^9^ Department of Earth System Science, Faculty of Science, Fukuoka University, Fukuoka, Japan

**Keywords:** TCA cycle, mtPDC, auxin transport, PIN, NPH3-like, ROS, root development

## Abstract

The mitochondrial pyruvate dehydrogenase complex (PDC) plays a crucial role in linking the glycolysis pathway and the tricarboxylic acid (TCA) cycle. Previously, we reported that a mutation of *MAB1*, encoding an E1β subunit of PDC, affects the abundance of auxin efflux carriers PIN-FORMED proteins (PINs) via reduced recycling and enhanced degradation in vacuoles. Here, we further analyzed the effects of TCA cycle inhibition on vesicle trafficking using both the *mab1-1* mutant and 3-BP, a TCA cycle inhibitor. Pharmacological and genetic impairment of the TCA cycle induced the aggregated components of ARA6, which is a plant-unique RAB5 GTPase that mediates endosomal trafficking to the plasma membrane. In addition, MAB4, which is an NPH3-like protein that inhibits PIN internalization from the plasma membrane, was severely reduced in 3-BP-treated roots and *mab1-1*. Furthermore, TCA cycle impairment led to the accumulation of reactive oxygen species in root tips, and treatment with H_2_O_2_ reduced MAB4 levels while increasing the internalization of PIN2 from the plasma membrane, and aggregated ARA6-positive compartments. These results suggest that TCA cycle impairment targets PIN proteins for degradation in the vacuole by disrupting both the MAB4-mediated block of internalization and the ARA6-mediated endocytic pathway.

## 1 Introduction

Mitochondrial pyruvate dehydrogenase complex (mtPDC) is one of the most pivotal enzymatic machineries, linking glycolysis and the tricarboxylic acid (TCA) cycle. This multienzyme complex, which contains E1, E2, and E3 components, oxidatively decarboxylates pyruvate to NADH and acetyl-CoA, initiating the TCA cycle in mitochondria. The E1 component of mtPDC consists of a catalytic α subunit and a regulatory β subunit. In the model plant *Arabidopsis thaliana*, *IAA-CONJUGATE-RESISTANT 4* (IAR4; *At1g24180*) and *IAR4-LIKE* (*IAR4L*; *At1g59900*) encode two E1α homologs (LeClere et al., 2004; Quint et al., 2009; Luethy et al., 1995), and *MACCHI-BOU 1* (*MAB1*; *At5g50850*) encodes an E1β subunit of mtPDC (Ohbayashi et al., 2019; Luethy et al, 1994) The E2 component of mtPDC, encoded by three genes *mtE2-1* (*At3g52200*), *mtE2-2*(*At3g13930*), and *mtE2-3*(*At1g54220*), is responsible for converting decarboxylated pyruvate into coenzyme A (Guan et al., 1995; Thelen et al., 1999; Taylor et al., 2004). Notably, mtE2-1 and mtE2-2 each contain two lipoic acid domains, whereas mtE2-3 has only one (Song and Liu, 2015). The E3 component, which is primarily involved in reducing coenzyme NAD^+^, is encoded by the nuclear *mitochondrial LIPOAMIDE DEHYDROGENASE1* (*mtLPD1*; *At1g48030*) and *mtLPD2* (*At3g17240*) (Lutziger and Oliver, 2001).

The contribution of mtPDC to plant development has been demonstrated in studies using *A. thaliana* mutants. Arabidopsis E1 mutants *iar4* and *mab1* exhibit defects in primary root growth and root hair emergence (LeClere et al., 2004; Quint et al., 2009; Ohbayashi et al., 2019). E2 mutants have defects in plant development (Yu et al., 2012; Song and Liu, 2015). Mutations in *mtE2-1* reduce organ size, and knockout of *mtE2-2* causes an embryo-lethal phenotype. In addition, disruption of *mtLPD2* more profoundly inhibits root growth, including primary and lateral roots, and decreases seedling size compared to wild-type seedlings, especially in the presence of arsenate (Chen et al., 2014). The phytohormone auxin plays a critical role in plant organ development, including cell division, cell elongation, and cell differentiation (Dhonukshe et al., 2007; Kleine-Vehn et al., 2008). Therefore, mtPDC likely contributes to organ development by modulating auxin action. In fact, the *iar4* mutant has been reported to exhibit auxin-related phenotypes, including auxin resistance and reduced auxin response, which were rescued by increasing the endogenous auxin levels (Quint et al., 2009). These results suggest that mtPDC is involved in auxin homeostasis. The *mab1* mutation has also been shown to severely reduce the level of auxin efflux carriers in the plasma membrane, which drive intercellular auxin transport essential for organ development (Ohbayashi et al., 2019). Thus, PDC could contribute to auxin-regulated root development by modulating polar auxin transport. Whereas impaired mtPDC could affect several aspects of auxin action in root development, the molecular links between mtPDC and auxin remain undetermined.

The TCA cycle is one of the main sources of reactive oxygen species (ROS) within plant cells (Møller, 2001; Fleury et al., 2002; Rhoads et al., 2006; Mao et al., 2018). High-energy electrons produced by the TCA cycle, such as NADH and FADH_2_, can lead to the generation of ROS, including superoxide anion (O_2_^•-^), hydrogen peroxide (H_2_O_2_), and hydroxyl radicals (^•^OH), when transferred through the mitochondrial electron transport chain. Notably, succinate dehydrogenase (SDH) contributes to mitochondrial ROS production in both animals and plants (Gleason et al., 2011; Quinlan et al., 2012; Jardim-Messeder and Moreira-Pacheco, 2016). SDH generates ROS in both the forward reaction, with electrons supplied by succinate, and the reverse reaction, with electrons supplied by the reduced ubiquinone pool in rat skeletal muscle (Quinlan et al., 2012). In Arabidopsis, a point mutation at the substrate-binding site of SDH1-1 decreases SDH activity and reduces the generation of mitochondrial ROS and root cell ROS in response to salicylic acid (Gleason et al., 2011). In addition, mutations in *IAR4*, encoding Arabidopsis mtPDC E1α, lead to increased ROS accumulation in root tips under salt stress (Fu et al., 2019). Furthermore, exogenously applied intermediate metabolites in the TCA cycle alter ROS levels in Arabidopsis root tips (Zhang et al., 2023). Succinate application increases H_2_O_2_ levels, whereas alpha-ketoglutarate and citrate applications decrease H_2_O_2_ levels. These results indicate a close association between ROS levels and TCA cycle activity in Arabidopsis roots. ROS, with their high chemical reactivity, play crucial roles in cellular physiological functions and redox balance. In root development, ROS act as signaling molecules regulating the formation of root structure and morphology, as well as the identity, state, and maintenance of stem cells, and the transition from proliferation to differentiation in the root (Tsukagoshi et al., 2010; Yu et al., 2016). Thus, ROS could serve as a link between the TCA cycle and auxin-regulated root development.

Our previous study showed that in *mab1-1*, a weak mutant allele of *MAB1* encoding mtPDC E1β, the amount of PIN auxin efflux carrier proteins was severely reduced compared to the wild type, likely contributing to the observed auxin-related phenotypes in this mutant (Ohbayashi et al., 2019). Here, we performed further analyses to investigate the effects of TCA cycle impairment on PIN trafficking using the *mab1-1* mutation and the pyruvate analog 3-bromopyruvate (3-BP), which inhibits glycolysis and the TCA cycle. Components localizing the plant-unique RAB5 GTPase ARA6, involved in the endocytic pathway from endosomes to the plasma membrane, were aggregated in 3-BP-treated roots and *mab1-1* mutants. Pharmacological and genetic impairment of the TCA cycle severely reduced the localization of the NPH3-like protein MAB4, which specifically inhibits PIN internalization. These results suggest that TCA cycle impairment could cause PIN proteins to be relocated for degradation by promoting internalization from the plasma membrane and blocking the endosomal recycling pathway to the plasma membrane. In addition, we found that TCA cycle impairment induced ROS accumulation in root tips, and an increase in ROS levels promoted MAB4 reduction, PIN2 internalization, and ARA6 aggregation. These data suggest that TCA cycle impairment induces ROS accumulation, leading to PIN2 internalization and degradation via reduced MAB4 levels and the aggregation of ARA6 components in Arabidopsis roots.

## 2 Materials and methods

### 2.1 Plant materials and growth conditions

*Arabidopsis thaliana* accession Columbia (Col) was used as the wild type. The following mutant alleles were used: *mab1-1* (Ohbayashi et al., 2019), *mel1-1 mel2-1 mel3-1 mel4-1* (Furutani et al., 2011). The transgenic lines *PIN2-GFP* (Xu and Scheres, 2005), *35S:MAB4-GFP* (Furutani et al., 2011), *35S:ST-mRFP* (Latijnhouwers et al., 2005), *SYP43p:mRFP-SYP43*, *ARA6p:gARA6-mRFP*, *ARA7p:mRFP-gARA7* (Ebine et al., 2011), and *CLCp: CLC-mKO* (Ito et al., 2012) were described previously. *35S:secRFP* (4B8-2; Zheng et al., 2005) and *UBQ10p:YFP-PIP1;4* (Wave 138Y; Geldner et al., 2009) were obtained from the Nottingham Arabidopsis Stock Center (https://arabidopsis.info). *PIN2p:PIN2-mCherry* was generated by replacing the *GFP* fragment of the *PIN2p:PN2-GFP* construct (Xu and Scheres, 2005) with *mCherry*. Seeds were surface-sterilized, plated on half strength Murashige and Skoog (1/2 MS) medium plates, and germinated as described previously (Furutani et al., 2004). Plants were transferred to soil and grown at 23°C under constant light as described previously (Ohbayashi et al., 2019).

### 2.2 3-BP and H_2_O_2_ treatment and microscopy

1 mM 3-BP or 2 mM H_2_O_2_ was exogenously applied by incubating the seedlings of *PIN2p:PIN2-GFP*, *PIN2p:PIN2-mCherry*, *PIN2p:PIN2-mCherry*/*35S:MAB4-GFP*, *35S:MAB4-GFP*, or *YFP-PIP1;4* in 1/2 MS liquid medium for 90 min, or 60 min, respectively. At 45 min before microscopic observation, 7.5 μM FM4-64 was applied. For the uptake of FM4-64, we applied 7.5 μM FM4-64 in 1/2 MS liquid medium for the indicated times. Images were obtained using a Leica SP8x, Olympus SpinSR, and Zeiss LSM980 confocal microscope.

### 2.3 ROS staining in Arabidopsis roots

To detect superoxides, nitroblue tetrazolium (NBT) staining was conducted as described previously (Yang et al., 2014). Five-day-old seedlings were stained with 0.05% NBT in 0.1 M sodium phosphate (NaPi) buffer, pH 7.1, for 30 min and then destained with 70% ethanol. The samples were then cleared with an 8:1:2 (w/v/v) mixture of 2,2,2-trichloroethane-1,1-diol, glycerol, and water before observation under a Nikon Ni-U microscope equipped with Nomarski optics. For detecting H_2_O_2_, 3,3’-diaminobenzidine (DAB) staining was conducted as described previously (Guan et al., 2019). Seedlings were incubated in 0.5 mg/mL DAB in 50 mM Tris–HCl, pH 5.0, for 2 h, washed three times with water, and observed using a Nikon Ni-U microscope. To detect ROS in the Arabidopsis root, 5-day-old Col seedlings were incubated in darkness in 1/2 MS liquid medium containing 0.5 mM 2’,7’-Dichlorofluorescein diacetate (DCFH-DA) for 30 min, followed by incubation in 1/2 MS liquid medium containing 100 μM DCFH-DA for 30 min, and observed using an Olympus SpinSR confocal microscopy.

### 2.4 3-BP, H_2_O_2_, and GSH treatments of Arabidopsis roots

For the 3-BP treatment assay, the seeds of Col, *PIN2p:PIN2-mCherry*, *PIN2p:PIN2-mCherry*/*35S:MAB4-GFP*, *PIN2p:PIN2-GFP*, and *PIN2p:PIN2-GFP*/*mel1-1 mel2-1 mel3-1 mel4-1* were sown directly on MS medium appropriately supplemented with an equivalent amount of solvent (DMSO or ethanol), 0.1 mM, 0.5 mM, or 1 mM 3-BP. For the H_2_O_2_ treatment assay, the seeds of Col-0 and *mab1-1* were sown directly on MS or MS medium supplemented with 2 mM H_2_O_2_. The plates were placed vertically in a light incubator for 5 days before being photographed. For the GSH treatment assay, 5-day-old seedlings of Col-0 and *mab1-1* were transferred to MS or MS medium supplemented with 1 mM GSH. The root tips were placed in a straight line. The plates were oriented vertically in a light incubator for another 3 days before they were photographed. In addition, *mab1-1* was sown on MS or MS medium supplemented with 1 mM GSH for 8 days before being photographed.

## 3 Results

### 3.1 3-BP treatment phenocopied the effects of *mab1* mutations on root growth

**Figure 1:**
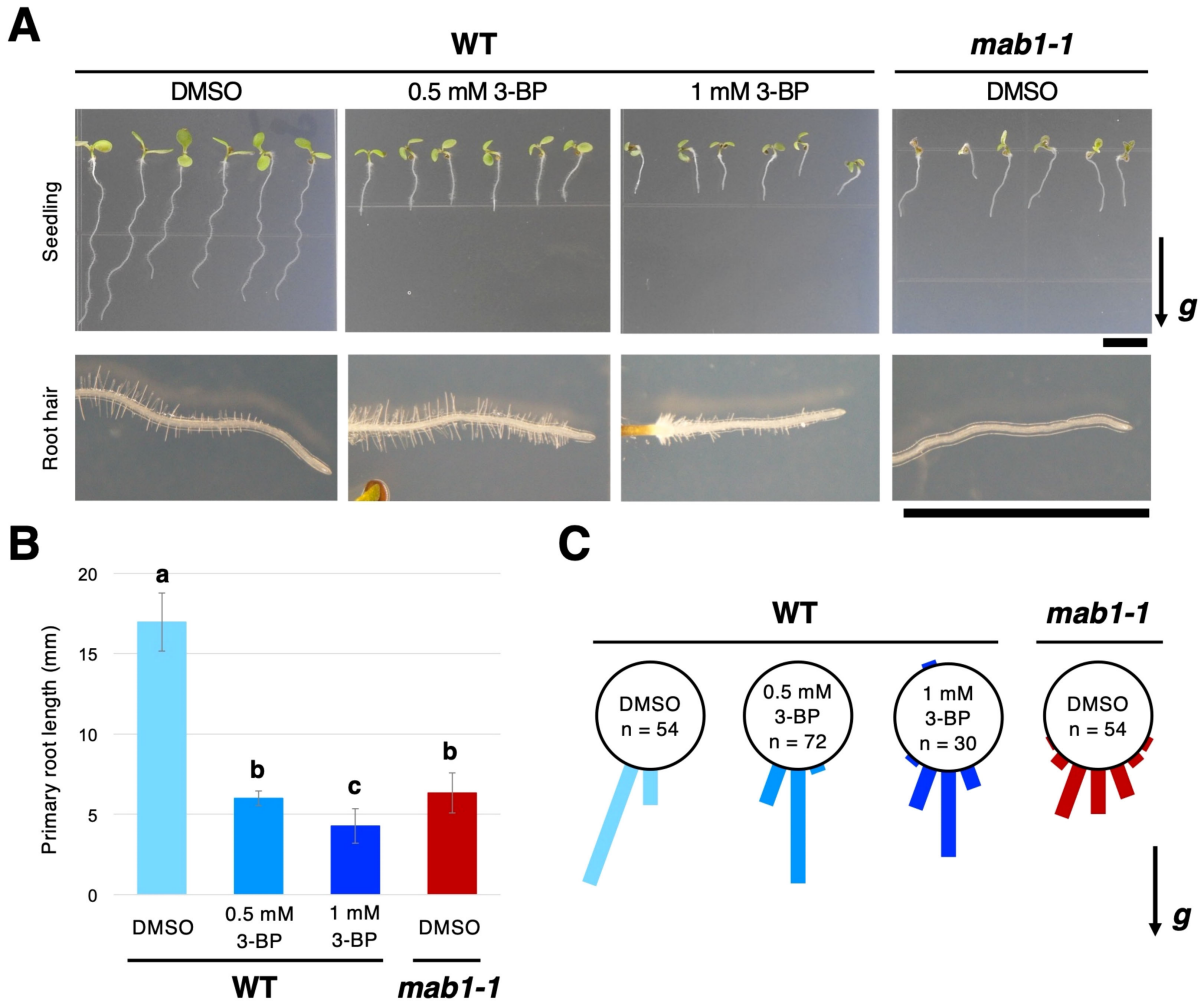
Treatment with 3-BP phenocopied the effects of the *mab1* mutation on Arabidopsis seedlings. **(A)** The 5-day-old seedlings and root hair development of wild-type (WT) Col treated with 3-BP (0.5 mM and 1 mM, respectively), and the *mab1-1* mutant. **(B, C)** Primary root length **(B)** and primary root growth direction **(C)** of the 5-day-old Col treated with 3-BP and *mab1-1*. Different letters above the bars indicate statistically significant difference at P < 0.01 (two-tailed Student's *t*-test). The arrow marked with “*g*” represents the direction of gravity in **(A, C)**. Scale bars = 5 mm.

Previously, it has been reported that *mab1* mutations disrupt auxin-regulated root growth, including root elongation, root hair growth, and root growth direction, along with the reduced activity of PDH linking glycolysis and the TCA cycle (Ohbayashi et al., 2019). Here, we examined the effects of 3-BP, an inhibitor of glycolysis and the TCA cycle, on Arabidopsis root growth. 3-BP is a pyruvate mimetic that represses the activity of several enzymes in the glycolysis pathway and the TCA cycle (Shoshan, 2012; Jardim-Messeder and Moreira-Pacheco, 2016). When treated with 0.5 or 1 mM 3-BP, the primary root growth of 5-day-old seedlings was severely repressed in a dose-dependent manner, and root hair formation was also inhibited at 1 mM 3-BP (**Figure 1A**), resembling the phenotypes observed in the *mab1-1* mutant (**Figures 1A, B**). Furthermore, this inhibitor disturbed the growth direction of the primary roots. For 3-BP treatment, primary root growth varied more widely in direction angle than for DMSO treatment (**Figure 1C**). These results suggest that treatment with 3-BP has similar effects to the *mab1-1* mutation on Arabidopsis root growth, indicating that the phenotypes observed under the 3-BP condition are reflective of TCA cycle impairment.

### 3.2 3-BP application induced the internalization of PIN2 from the plasma membrane

**Figure 2:**
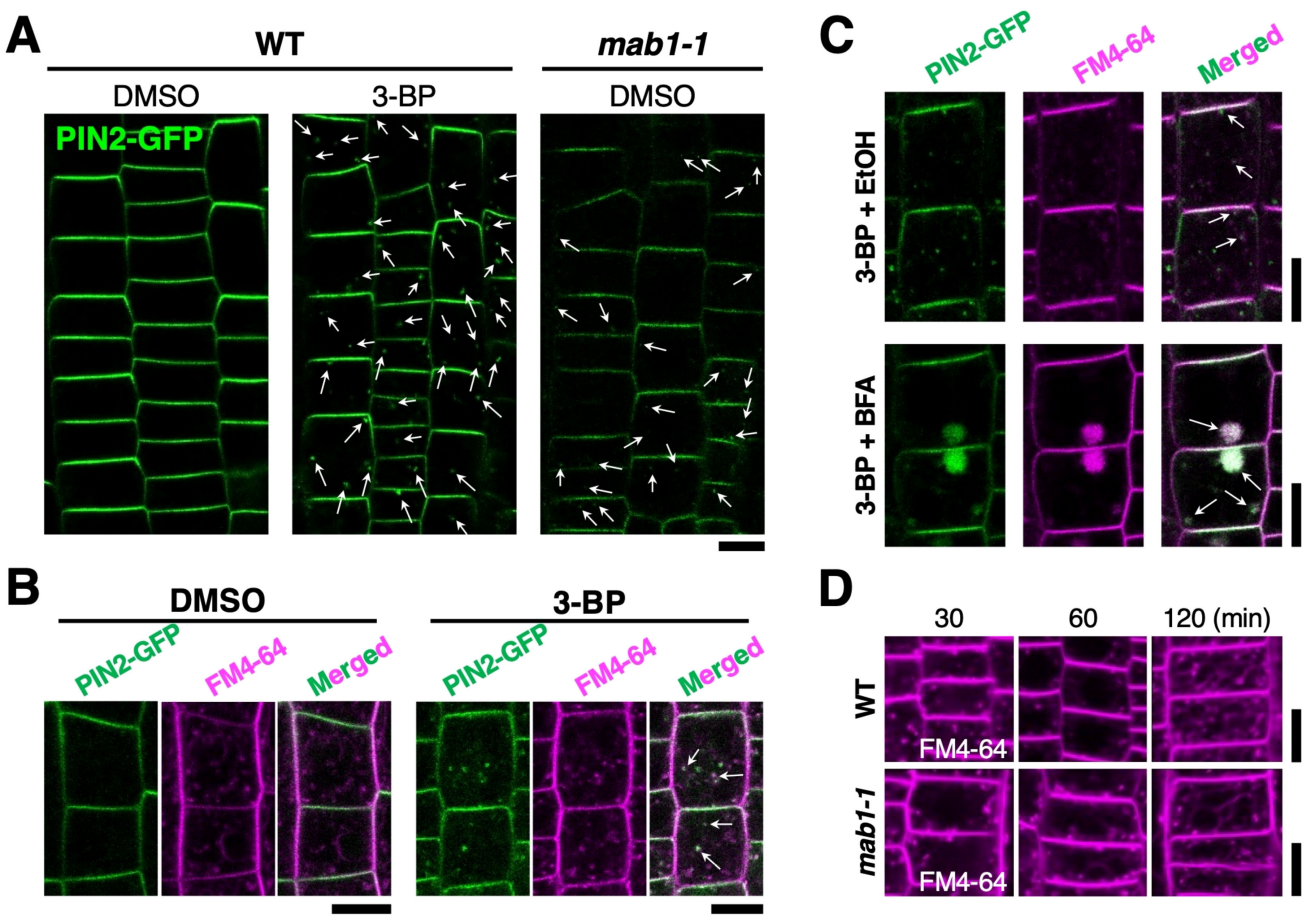
3-BP promoted PIN2 endocytosis without affecting the vacuolar targeting pathway.**(A)** PIN2-GFP localization in wild-type Col (WT) and *mab1-1* roots harboring *PIN2p:PIN2-GFP* treated with DMSO and 0.5 mM 3-BP. Arrows indicate internalized PIN2-GFP signals. **(B)** Confocal images of the fluorescence of GFP (green, left) and FM4-64 (magenta, middle) in the epidermal cells of 5-day-old wild-type seedlings expressing PIN2-GFP. Arrows demonstrate co-labeling with punctate signals of GFP and FM4-64 (right). **(C)** Internalized PIN2-GFP (top) aggregated into BFA compartments in the epidermal cells of 5-day-old seedlings treated with 0.5 mM 3-BP (bottom). Arrows show co-labeling with GFP and FM4-64 (right). **(D)** FM4-64 uptake time course in the epidermis of 5-day-old wild-type (top) and *mab1-1* (bottom) seedlings. Scale bars = 10 μm.

A recent study has shown that the *mab1* mutation accelerates PIN degradation in vacuoles and reduces PIN recycling via endosomes (Ohbayashi et al., 2019). Therefore, we examined the alteration of PIN2-GFP trafficking in more detail in the 3-BP condition. 3-BP treatment reduced the level of PIN2-GFP in the plasma membrane (**Figure 2A**; **Supplementary Figure S1**). A number of punctate signals of PIN2-GFP were also induced in the cytosol of epidermal cells of 3-BP-treated wild-type roots, similar to *mab1-1* roots, compared with the control (**Figure 2A**; **Supplementary Figure S1**). In addition, the *mab1-1* mutation was found to reduce the sensitivity of PIN2-GFP to 3-BP, indicating that 3-BP affected PIN2-GFP localization through the *MAB1* pathway as expected (**Supplementary Figure S1A, B**). Next, to test whether intracellular PIN2-GFP localizes to the endocytic pathway, we stained 3-BP-treated roots with the fluorescent endocytic tracer FM4-64. Internalized FM4-64 hardly overlapped with the PIN2-GFP in DMSO-treated wild-type roots, whereas intracellular punctate GFP signals often colocalized with FM4-64 in 3-BP-treated roots (**Figure 2B**). In addition, when co-treated with BFA, an inhibitor of intracellular vesicle trafficking, 3-BP-induced PIN2-GFP punctate signals in BFA-induced compartments were co-stained with FM4-64 (**Figure 2C**). These results indicate that 3-BP treatment induces the internalization of PIN2-GFP from the plasma membrane via an endosomal pathway. Next, to check the effects of TCA cycle impairment on the endosomal pathway, *mab1-1* roots were stained with FM4-64 for *in vivo* tracking of the endosomal pathway. Under normal conditions, FM4-64 was taken up into the vacuolar membrane via endosomes and PVCs after 1 h of treatment in epidermal cells of *mab1-1* roots (**Figure 2D**). Two hours after treatment, FM4-64 was accumulated more prominently in the vacuolar membrane in both backgrounds (**Figure 2D**). There was no difference in the time course of endocytosis and vacuolar targeting between the wild type and *mab1-1*. The shape of the vacuole was also unaffected by the *mab1-1* mutation (**Figure 2D**; **Supplementary Figure S2**). These results indicate that TCA cycle impairment could induce PIN2 internalization without affecting endocytosis or vacuolar targeting pathways.

### 3.3 TCA cycle impairment affected the localization of ARA6, involved in endosomal recycling

**Figure 3:**
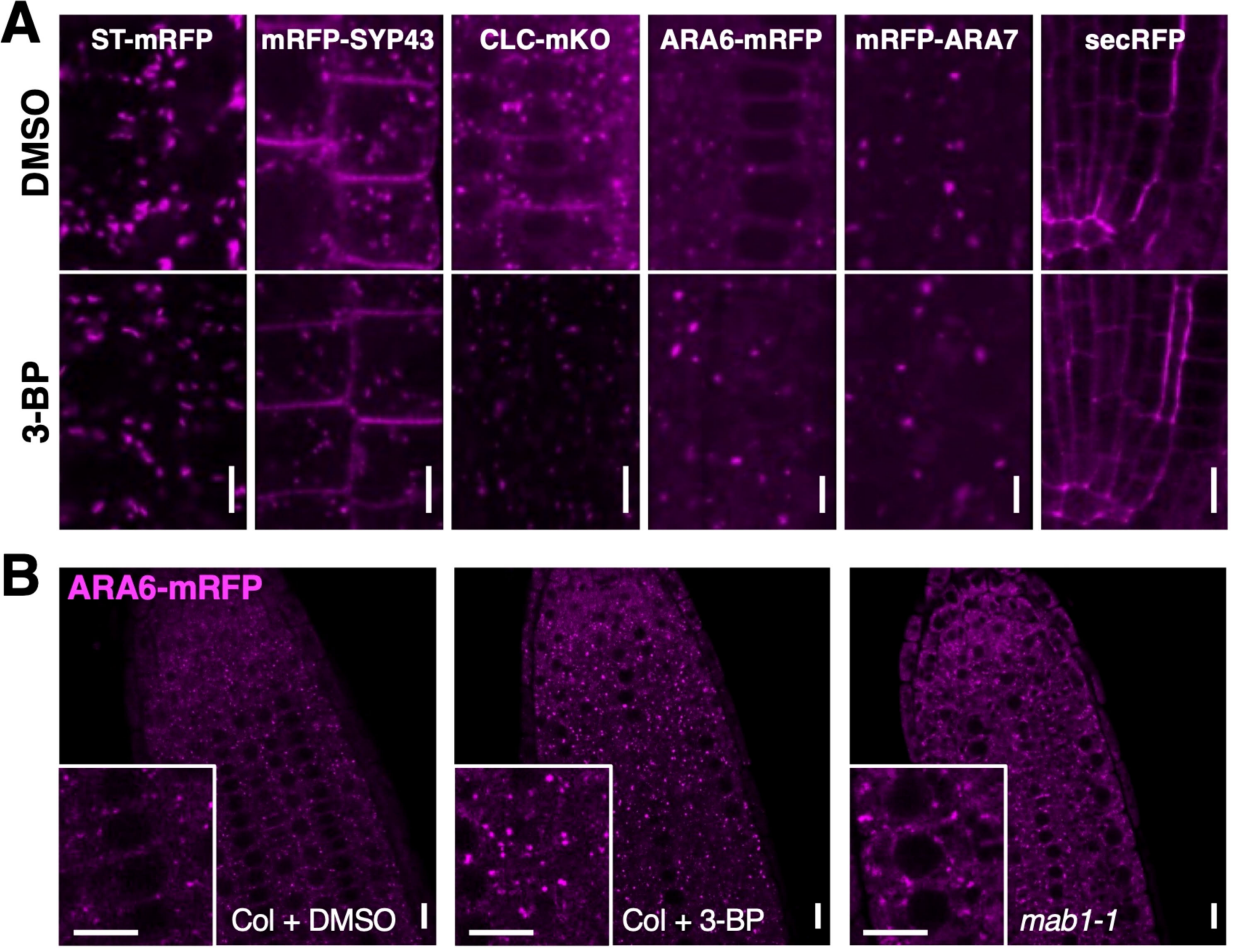
TCA cycle impairment affected ARA6 localization.**(A)** Confocal images of endomembrane markers, ST-mRFP for Golgi, mRFP-SYP43 for TGN and PM, CLC-mKO for TGN, ARA6-mRFP for late endosome, mRFP-ARA7 for early endosome, and a secretion marker SEC-RFP, in DMSO- and 3-BP-treated roots of 5-day-old seedlings. **(B)** ARA6-mRFP localization in root tips of DMSO- (left) and 3-BP-treated wild-type Col (middle) and *mab1-1* (right) seedlings. Insets are enlarged figures of respective images. Scale bars = 10 μm.

To examine the effect of TCA cycle impairment on endomembrane trafficking in more detail, endomembrane and vesicle trafficking markers were used for localization analysis in 3-BP-treated roots. ST-mRFP for the Golgi apparatus, mRFP-SYP43 and CLC-mKO for the trans-Golgi network (TGN), ARA6-mRFP for endosomal recycling, mRFP-ARA7 for endosomal/vacuolar trafficking, and secRFP for the secretion pathway were used (**Supplementary Figure S3**). 3-BP treatment did not affect the localization of ST-mRFP, mRFP-ARA7, or secRFP, whereas reductions in mRFP-SYP43 and CLC-mKO and aggregation of ARA6-mRFP were observed in 3-BP-treated roots (**Figure 3A**). However, the signals of ST-mRFP, mRFP-ARA7, mRFP-SYP43 and CLC-mKO were not reduced by the *mab1-1* mutation (**Supplementary Figures S4**, **S5**). Only the localization of ARA6-mRFP was affected in both 3-BP-treated and *mab1-1* roots (**Figure 3B**). 3-BP treatment and the *mab1-1* mutation induced the aggregation of ARA6-mRFP signals in root cells. ARA6 is a plant-unique RAB5 GTPase that regulates an endosomal recycling pathway to the plasma membrane (Ueda et al., 2001; Ebine et al., 2011; Bottanelli et al., 2011, Bottanelli et al., 2012). These data collectively suggest that TCA cycle impairment could reduce endosomal recycling.

### 3.4 3-BP treatment internalized PIN2 through the reduction of MAB4 in the plasma membrane

**Figure 4:**
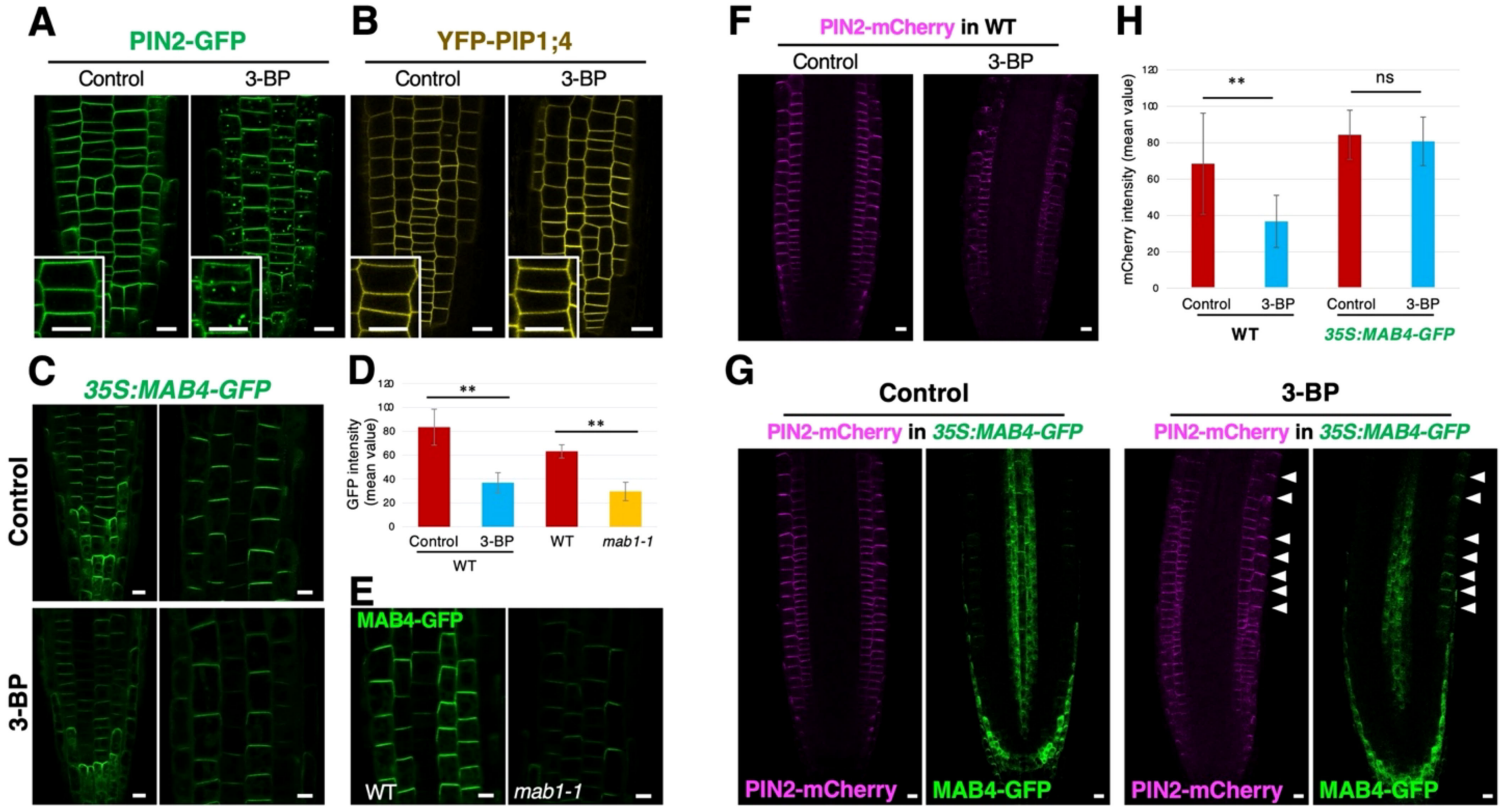
3-BP treatment reduced the localization of MAB4, a specific regulator of PIN localization, leading to the decrease of the PIN2 level in the plasma membrane. **(A, B)** The localization of PIN2-GFP **(A)** and YFP-PIP1;4 **(B)** in root epidermal cells of DMSO- (left) and 3-BP-treated (right) 5-day-old seedlings harboring *PIN2p:PIN2-GFP* and *UBQ10p:YFP-PIP1;4* (Wave138Y), respectively. Insets are enlarged images of respective figures. Arrows indicate internalized signals of PIN2-GFP. **(C)** MAB4-GFP localization in DMSO- (top) and 3-BP-treated (bottom) roots of 5-day-old seedlings harboring *35S:MAB4-GFP*. **(D)** Comparison of the GFP signal intensity in the plasma membrane of root epidermis of 5-day-old *35S:MAB4-GFP* seedlings treated with DMSO and 3-BP, and of wild-type (WT) and *mab1-1* seedlings. Values and bars represent means ± SD of three biological replicates, and differences between the means were assessed for statistical significance using Student’s *t*-test (**P < 0.01). **(E)** MAB4-GFP localization in the root epidermis of 5-day-old WT (left) and *mab1-1* (right) seedlings harboring *35S:MAB4-GFP*. **(F)** Confocal images of PIN2-mCherry in ethanol- (left) and 3-BP-treated (right) root tips of 5-day-old seedlings harboring *PIN2p:PIN2-mCherry*. **(G)** Confocal images of MAB4-GFP (green) and PIN2-mCherry (magenta) in ethanol- (left) and 3-BP-treated (right) root tips of 5-day-old seedlings harboring *35S:MAB4-GFP* and *PIN2p:PIN2-mCherry*. Arrowheads indicate the plasma membrane of epidermis where MAB4-GFP was localized. **(H)** Comparison of the mCherry signal intensity in the plasma membrane of root epidermis of 5-day-old *PIN2p:PIN2-mCherry* seedlings treated with ethanol and 3-BP in the WT and *35S:MAB4-GFP* background. In the *35S:MAB4-GFP* background, the mCherry signals were measured only in the plasma membrane where MAB4-GFP was detected. Values and bars represent means ± SD of three biological replicates, and differences between the means were assessed for statistical significance using Student’s *t*-test (**P < 0.01, ns means no significant differences). Comparison of the Scale bars = 10 μm.

To determine whether the effect of 3-BP treatment is specific to PIN proteins, the localization of another membrane protein, PIP1;4, was analyzed after 3-BP treatment. Under normal conditions, YFP-PIP1;4 was localized in the plasma membrane of epidermal cells of 3-BP-treated roots, although 3-BP application induced punctate signals of PIN2-GFP in the cytosol and reduced PIN2-GFP levels in the plasma membrane (**Figures 4A, B**; **Supplementary Figure S6**). These results indicate that 3-BP significantly affects PIN2 localization but not PIP1;4 localization. NPH3-like proteins MAB4 and MELs have been previously shown to specifically regulate PIN localization by inhibiting the internalization of PIN proteins from the plasma membrane (Furutani et al., 2004, Furutani et al., 2011). These proteins are suggested to be targets of 3-BP in the internalization of PIN proteins. To confirm this, the effect of 3-BP treatment on MAB4 localization was analyzed in *35S:MAB4-GFP* expressing roots. MAB4-GFP was localized in the plasma membrane in a polarized manner similar to PIN proteins under normal conditions, whereas 3-BP application severely reduced the signals of MAB4-GFP in the plasma membrane (**Figures 4C, D**). In addition, we observed the same effects of the *mab1* mutation on MAB4 localization. MAB4-GFP signals were severely reduced in *mab1-1* roots compared to wild-type roots (**Figures 4D, E**; **Supplementary Figure S7**). Next, co-localization of MAB4-GFP and PIN2-mCherry was analyzed in 3-BP-treated roots harboring both *35S:MAB4-GFP* & *PIN2p:PIN2-mCherry* to examine the link between 3-BP-induced MAB4 reduction and PIN2 internalization (**Supplementary Figure S8**). Under normal conditions, MAB4-GFP and PIN2-mCherry were co-localized in the upper side of the plasma membrane of epidermal and lateral root cap cells (**Supplementary Figure S8A**). In 3-BP-treated roots, MAB4-GFP and PIN2-mCherry were severely reduced (**Supplementary Figure S8B**). PIN2-mCherry was internalized from the plasma membrane and reduced in the plasma membrane in a few cells where the expression of MAB4-GFP was lost or severely decreased. In contrast, cells with stronger expression of MAB4-GFP in the plasma membrane tended to display a stronger level of PIN2-mCherry in the plasma membrane. Our results indicate a close correlation between MAB4 reduction and PIN2 internalization in the presence of 3-BP. Next, to investigate whether overexpression of *MAB4* could suppress 3-BP-induced internalization of PIN2, the sensitivity of PIN2-mCherry to 3-BP treatment was compared between the wild-type and MAB4 overexpression background (**Figures 4F, H**). 3-BP treatment decreased the level of PIN2-mCherry in wild type, whereas 3-BP treatment did not affect the level of PIN2-mCherry in cells where MAB4-GFP remained in the plasma membrane after 3-BP treatment. Overexpression of MAB4-GFP also reduced the sensitivity of root growth to 3-BP treatment (**Supplementary Figure S9**). In contrast, mutations of other *MAB4* family member genes, *MAB4*/*ENP*/*NPY1-LIKE*s (*MEL*s) (Furutani et al., 2011), increased sensitivity of PIN2-GFP and root growth to 3-BP (**Supplementary Figure S10**). These results indicate that 3-BP treatment internalizes PIN2 through the reduction of NPH3-like proteins in the plasma membrane.

### 3.5 TCA cycle impairment increased the ROS level

**Figure 5:**
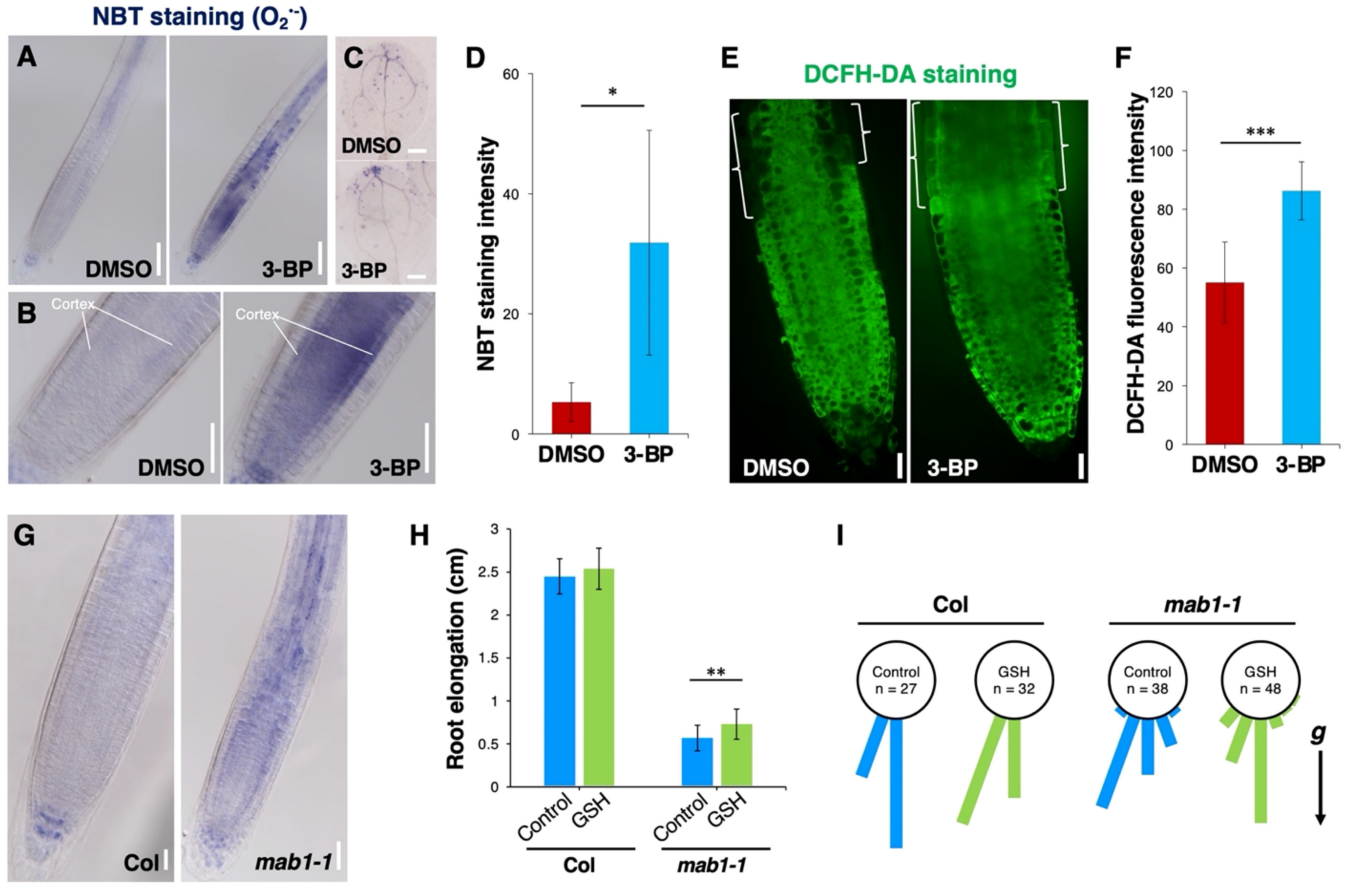
TCA cycle impairment increased ROS level in root tips. **(A–C)** NBT staining for superoxide in the primary root **(A, B)** and cotyledon **(C)** of DMSO- and 3-BP-treated 5-day-old seedlings. **(B)** Enlarged images of figures in **(A)**. **(D)** Comparison of the NBT staining intensity in Col roots treated with DMSO and 3-BP (n = 5). Error bars represent ± SD. Asterisks indicate statistically significant differences as determined by Student’s *t*-test (*P < 0.05). **(E)** DCFH-DA staining in the primary root of 5-day-old wild-type Col treated with DMSO (left) and 3-BP (right). Brackets indicate the transition zone of the Col root. **(F)** Comparison of the DCFH-DA fluorescence intensity in the cytoplasm of the transition zone of Col roots treated with DMSO and 3-BP (n ≥ 10). Error bars represent ± SD. Asterisks indicate statistically significant differences as determined by Student’s *t*-test (***P < 0.001). **(G)** NBT staining for superoxide in the primary root of Col and *mab1-1* 5-day-old seedlings. **(H)** Comparison of primary root elongation of 5-day-old seedlings of Col and *mab1-1* treated with GSH. Values and bars represent means ± SD of three biological replicates, and asterisks indicate a significant difference between the means at P < 0.01 (two-tailed Student’s *t*-test). **(I)** Growth direction of the primary root of 5-day-old seedlings of Col and *mab1-1* on medium containing GSH. The arrow marked with “*g*” represents the direction of gravity. Scale bars = 100 μm in **(A)**, 50 μm in **(B, G)**, 200 μm in **(C)**, and 20 μm in **(E)**.

Mitochondrial respiration synthesizes ATP via the TCA cycle and also involves ROS homeostasis (Møller, 2001; Fleury et al., 2002; Rhoads et al., 2006; Mao et al., 2018). Recently, loss-of-function of *IAR4*, encoding a mitochondrial PDH E1α, has been reported to promote ROS accumulation under salt stress conditions (Fu et al., 2019). This raises the possibility that TCA cycle impairment promotes ROS production, leading to PIN internalization from the plasma membrane. To test this possibility, we determined the accumulation of ROS by NBT staining for superoxide radicals, by DAB staining for H_2_O_2_, and by the fluorescent probe DCFH-DA staining, in seedlings with an impaired TCA cycle. Under normal conditions, weak signals of NBT staining were found in columella cells, cortex cells, vascular tissues, and the tips of cotyledons in wild-type seedlings (**Figures 5A–C**). 3-BP application increased NBT staining in the tips of roots and cotyledons (**Figures 5A–D**). DAB staining was detected on the surface of the meristem and elongation regions, with weak staining around the quiescent center and vascular tissues (**Supplementary Figure S11A**). Conversely, 3-BP application increased the DAB staining both on the surface and inside cells in these regions (**Supplementary Figure S11A**). In addition, strong DCFH-DA fluorescence was detected in the transition zone of the root tip treated with 3-BP, compared with control (**Figures 5E, F**). In the *mab1-1* background, an increase in both NBT and DAB staining was detected over the entire root (**Figure 5G**; **Supplementary Figure S11B**). These results indicate that TCA cycle impairment could promote ROS accumulation. To investigate the effects of elevated ROS accumulation on root growth, wild-type and *mab1-1* seedlings were treated with H_2_O_2_. H_2_O_2_ application decreased the length of wild-type primary roots and disrupted the orientation of root growth similarly to *mab1-1* (**Supplementary Figures S11C–E**). The degree of H_2_O_2_-induced short root phenotypes in the wild type was higher than that in *mab1-1*. In contrast, reducing ROS by antioxidant GSH application partially suppressed the phenotypes of *mab1-1* roots (**Figures 5H, I**; **Supplementary Figure S12**). GSH-treated *mab1-1* roots elongated more than control *mab1-1* did, whereas GSH application did not affect wild-type root elongation. In addition, GSH treatment partially suppressed the phenotypes of root orientation in the *mab1-1* background. These results show that elevated ROS accumulation could contribute to the root phenotype of *mab1-1*.

### 3.6 H_2_O_2_ internalized PIN proteins through the reduction of MAB4 level in the plasma membrane

**Figure 6:**
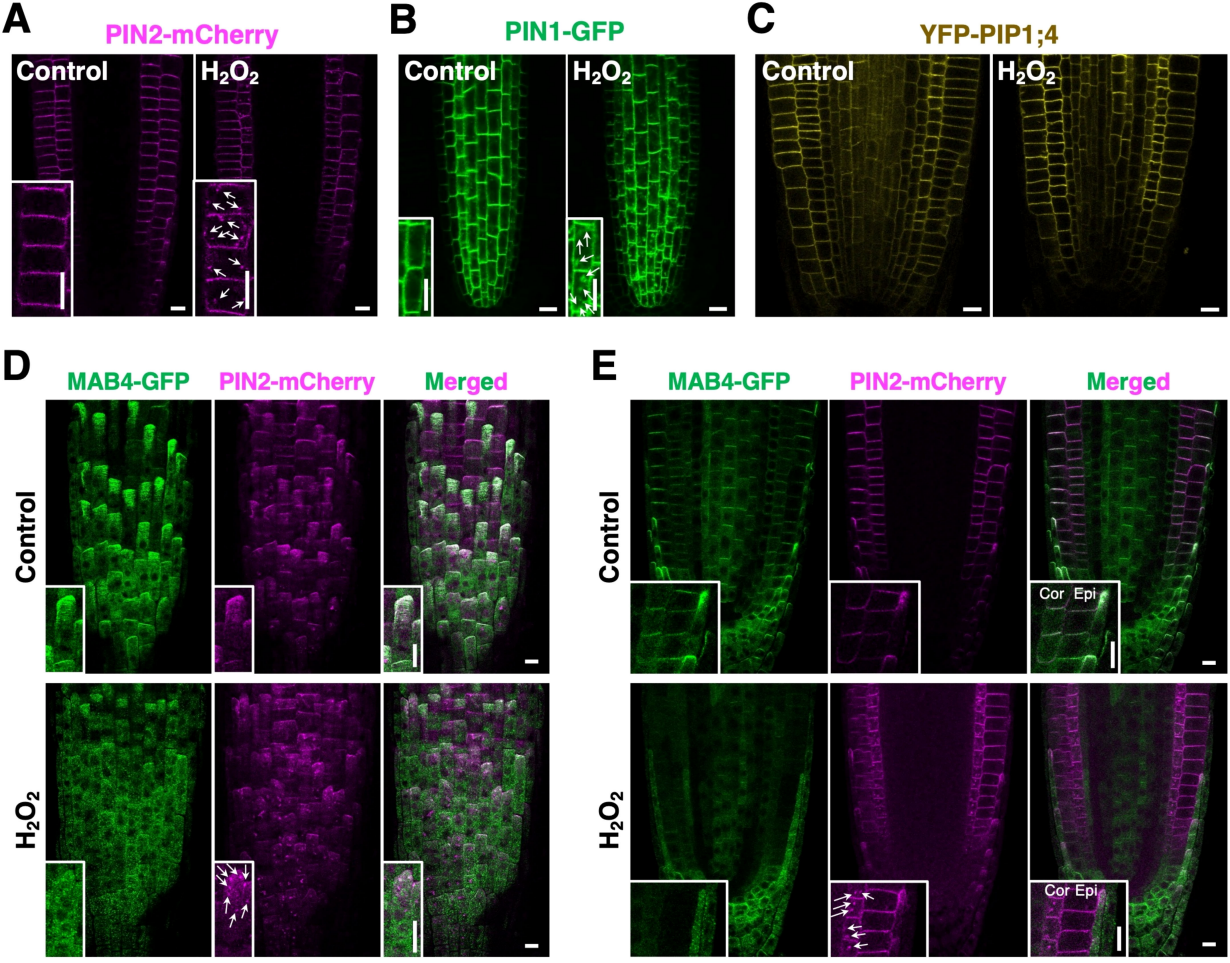
H_2_O_2_ disrupted MAB4 localization and promoted the internalization of PIN2. **(A–C)** The localization of PIN2-mCherry **(A)**, PIN1-GFP **(B)**, and YFP-PIP1;4 **(C)** in root epidermal cells of the control (left) and H_2_O_2_-treated (right) 5-day-old seedlings harboring *PIN2p:PIN2-mCherry*, *PIN1p:PIN1-GFP*, and *UBQ10p:YFP-PIP1;4* (Wave138Y), respectively. Insets are enlarged images of respective figures. Arrows in **(A, B)** indicate internalized signals of PIN2-mCherry and PIN1-GFP, respectively. **(D, E)** Confocal images of MAB4-GFP (left) and PIN2-mCherry (middle) on the surface **(D)** and cross-section **(E)** of the primary root tip of the 5-day-old wild-type seedlings harboring *35S:MAB4-GFP* and *PIN2p:PIN2-mCherry* treated with H_2_O_2_. Merged images of MAB4-GFP and PIN2-mCherry (right). Insets are enlarged images of respective figures. Cor, cortex; Epi, epidermis; LRC, lateral root cap. Scale bars = 10 μm.

ROS elevation is hypothesized to induce PIN internalization by reducing MAB4 levels in the plasma membrane. First, we analyzed the effects of elevated ROS accumulation on PIN localization to confirm this hypothesis. H_2_O_2_ application induced the internalization of PIN2-mCherry from the plasma membrane, particularly in the cortex cell layer (**Figure 6A**). Similarly, PIN1-GFP was notably internalized and aggregated in the vascular cells of H_2_O_2_-treated roots (**Figure 6B**). In contrast, H_2_O_2_ application did not affect the localization of YFP-PIP1;4 in root tips (**Figure 6C**; **Supplementary Figure S13A**), indicating a specific effect of elevated ROS on PIN localization. Next, we analyzed MAB4-GFP and PIN2-mCherry simultaneously in H_2_O_2_-treated roots harboring *35S:MAB4-GFP* and *PIN2p:PIN2-mCherry* to investigate the effect of ROS elevation on MAB4 localization. Under normal conditions, MAB4-GFP was co-localized with PIN2-mCherry in the plasma membrane of the lateral root cap, epidermis, and cortex, but in H_2_O_2_-treated roots, MAB4-GFP was localized in the cytoplasm while PIN2-mCherry was internalized (**Figures 6D, E**). In addition, ARA6-mRFP was aggregated by H_2_O_2_ treatment (**Supplementary Figure S13B**). These results indicate that H_2_O_2_ application promotes the internalization of PIN proteins from the plasma membrane by dissociating MAB4 from the plasma membrane and aggregation of ARA6.

## 4 Discussion

The mitochondrial TCA cycle functions in energy production, respiration, and primary metabolism, which significantly affects the growth and development of organisms. In Arabidopsis, mutations in the PDH complex, which plays a central role in controlling the entry of carbon into the TCA cycle, have been shown to cause defects in auxin-regulated organ development, resulting in severe reduction of auxin efflux carriers, specifically PIN proteins. However, the molecular link between the TCA cycle and PIN localization remains to be uncovered. Our results showed that TCA cycle impairment disrupted both the MAB4-mediated block of PIN internalization and the ARA6-mediated endocytic recycling pathway, resulting in a severe reduction of PIN levels in the plasma membrane. In addition, endogenous ROS levels were increased by TCA cycle impairment, and ROS elevation released the MAB4-blocked PIN internalization and disordered ARA6 localization. These findings suggest a molecular link between the mitochondrial metabolic pathway and polar auxin transport in root development.

### 4.1 ARA6-mediated endocytic recycling pathway in TCA cycle-defective cells

Previously, the *mab1-1* mutation has been shown to reduce PIN2 recycling and promote PIN2 targeting to the vacuole, resulting in a significant reduction of PIN2 levels in the plasma membrane (Ohbayashi et al., 2019). Our localization analysis of organelle markers in TCA cycle-disrupted plants revealed that ARA6-localized compartments were specifically aggregated (**Figure 3**). ARA6 is a plant-unique RAB GTPase involved in the endocytic recycling pathway. Our results suggest that TCA cycle impairment could reduce the endocytic recycling of PIN proteins by the aggregation of ARA6-positive endosomes. The ARA6 pathway was previously reported to play a role in the salinity response. Loss of function of ARA6 reduced salt-stress tolerance, whereas overexpression of constitutively active ARA6 increased resistance to salinity stress (Ebine et al., 2011). In addition, constitutively active ARA6 aggregated in response to high concentrations of salt, indicating that salinity stress could reduce ARA6-mediated endocytic recycling by affecting ARA6 localization. Furthermore, TCA cycle-defective *iar4* mutants showed increased sensitivity to salt stress in root growth and elevated accumulation of sodium ions (Fu et al., 2019). These data suggest that the ARA6-mediated endocytic recycling pathway, reduced in *iar4* mutants, increases the level of SALT OVERLY SENSITIVE 1 (SOS1), a plasma membrane Na^+^/H^+^ antiporter, under salinity stress conditions. Given the reduced *SOS1* expression in *iar4* mutants (Fu et al., 2019), the TCA cycle could be involved in controlling SOS1 levels via transcriptional activation and the ARA6-mediated endocytic recycling pathway under salinity stress conditions. In contrast, *mab1* mutations reduce PIN levels without transcriptional changes in PIN genes (Ohbayashi et al., 2019), suggesting only posttranscriptional regulation, including the ARA6-mediated endocytic recycling pathway. However, TCA cycle impairment did not affect the level of all membrane proteins. The level and localization of PIP1;4 were not affected (**Figure 4B**), implying specific effects for each membrane protein in addition to reduced ARA6-mediated endocytic recycling, such as the reduction of the transcript level for *SOS1* and the promotion of targeting of PIN to the vacuole.

### 4.2 MAB4-blocking PIN internalization in TCA cycle-defective cells

NPH3-like proteins in the MAB4/MEL subfamily specifically control the level of PIN proteins in the plasma membrane, but not that of the other membrane protein LTI6b, by blocking PIN internalization (Furutani et al., 2011). In the mutant, PIN2 internalization and targeting to the vacuole were accelerated, resulting in a significant reduction in PIN2 levels. Our findings showed that defective TCA cycle activity severely decreased MAB4 levels in the plasma membrane. Recently, MAB4/MEL proteins have been shown to interact with PIN proteins in the plasma membrane (Glanc et al., 2021). This suggests that TCA cycle impairment could disrupt the interaction between MAB4/MELs and PINs, leading to the loss of the MAB4-dependent block of PIN internalization. Indeed, 3-BP treatment specifically promoted the internalization of PIN2 but not that of FM4-64 or PIP1;4. This is consistent with a previous report that the PIN2 degradation pathway, targeting the protein to the vacuole, was accelerated in *mab1-1*. In TCA cycle-defective cells, reduced MAB4 levels could promote the internalization of PIN2 specifically from the plasma membrane, while ARA6 aggregation could reduce endocytic recycling to the plasma membrane. These two coupled events contribute to the targeting of PIN2 to the vacuole and its subsequent degradation, thereby decreasing PIN2 levels in the plasma membrane (**Supplementary Figure S14**).

### 4.3 Function of the TCA cycle in root growth and development

The developing root consists of three distinct zones: the meristem zone, the elongation zone, and the differentiation zone, in order from the apex. Multiple TCA metabolites, which accumulate asymmetrically in the root tip along the developmental axis, have recently been reported to have noncanonical functions in root growth and development (Zhang et al., 2023). Succinate specifically accumulates in the meristem zone, whereas aconitate accumulates in the differentiation zones. Treatment of root tips with succinate expands the meristem region and promotes root hair growth, accompanied by the upregulation of H_2_O_2_ levels in the elongation and differentiation regions, resulting in longer roots. In contrast, aconitate treatment reduces meristem size, resulting in short roots. Given that mtPDC is involved in the initiation of the TCA cycle, mtPDC could function in root growth and development by controlling the level and distribution of TCA metabolites. Indeed, citrate levels were reduced in short roots of *mab1-1* (Ohbayashi et al., 2019). Treatment with citrate, which accumulates in the meristem region (Zhang et al., 2023), reduces H_2_O_2_ levels in the root tips, in addition to reducing meristem size and elongation of root hairs. This is consistent with our findings that H_2_O_2_ levels were upregulated in root tips harboring impaired mtPDC, where citrate levels were reduced (**Figure 5**). The decrease in citrate in *mab1-1* roots could increase H_2_O_2_ levels, thereby promoting the targeting of PINs from the plasma membrane to the vacuole. Therefore, the proper mtPDC, which catalyzes the conversion of pyruvate into acetyl-CoA and condenses with oxaloacetate to generate citrate (Pearce et al., 2013), could function in the reduction of H_2_O_2_ levels through the control of citrate levels in the root tips. This contributes to the proper level of PIN proteins in the plasma membrane, establishing appropriate auxin distribution in root tips. Thus, the TCA cycle could regulate root growth and development via TCA metabolites-mediated ROS distribution, followed by ROS-regulated auxin transport.

## 5 Conclusions

The mitochondrial TCA cycle functions in energy production, respiration, and primary metabolism. In Arabidopsis, mutations in the PDH complex, which plays a central role in linking the glycolysis pathway and the TCA cycle, have been shown to cause a severe reduction of auxin efflux carriers, specifically PIN proteins, by targeting them to the vacuole. However, the molecular link between the TCA cycle and PIN trafficking remains uncovered. Here, our results showed that TCA cycle impairment disrupted the localization of MAB4, a specific blocker of PIN endocytosis, resulting in the promotion of PIN internalization. In addition, the ARA6-mediated endocytic recycling pathway was disordered, which could target PIN proteins to the vacuole, leading to a severe reduction of PIN levels in the plasma membrane. Furthermore, endogenous ROS levels were increased by TCA cycle impairment, and ROS elevation disrupted MAB4 localization and disordered ARA6 localization. Our findings suggest a molecular link between the mitochondrial metabolic pathway and polar auxin transport in root development via the control of ROS levels.

## Data Availability

The original contributions presented in the study are included in the article/**Supplementary Materials**. Further inquiries can be directed to the corresponding author.
