# Supplementary material for: TCA cycle impairment leads to PIN2 internalization and degradation via reduced MAB4 level and ARA6 components in Arabidopsis roots
Source: Front Plant Sci. 2024 Dec 16;15:1462235. doi: 10.3389/fpls.2024.1462235 (PMC11686435; doi:10.3389/fpls.2024.1462235)
Supplement: Supplementary file 1 [file DataSheet1.pdf]

Supplementary Material

TCA cycle impairment leads to PIN2 internalization and degradation via reduced MAB4 level and ARA6 components in Arabidopsis roots

Xiaomin Song#, Iwai Ohbayashi#, Song Sun, Qiuli Wang, Yi Yang, Mengyuan Lu, Yuanyuan Liu, Shinichiro Sawa and Masahiko Furutani*

*** Correspondence:** Masahiko Furutani: ma-furut@fukuoka-u.ac.jp

1 Supplementary Figures

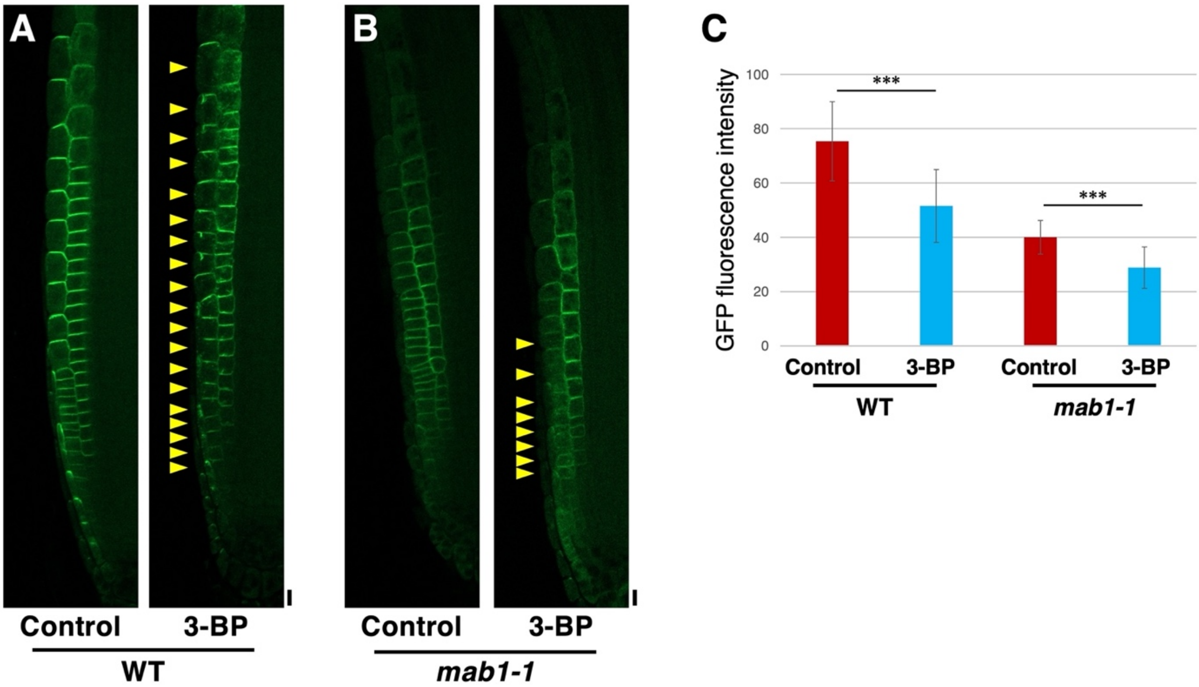

Supplementary Figure S1. Effect of 3-BP treatment on PIN2 localization in *mab1-1* roots

(**A**, **B**) PIN2-GFP localization in wild-type (WT) (**A**) and *mab1-1* (**B**) root tips of 5-day-old seedlings harboring *PIN2p:PIN2-GFP* (control: **left**, 3-BP treatment: **right**). Arrowheads indicate epidermal cells where punctate localization of PIN2-GFP was detected in 0%, 90% of WT roots treated with ethanol and 3-BP, and in ~12%, ~43% of *mab1-1* roots treated with ethanol and 3-BP, respectively. Scale bars = 10 µm. (**C**) Comparison of the GFP signal intensity in the plasma membrane of root epidermis of 5-day-old *PIN2p:PIN2-GFP* seedlings treated with ethanol and 3-BP in the WT and *mab1-1* background. Values and bars represent means ± SD of three biological replicates, and differences between the means were assessed for statistical significance using Student's *t*-test (***P < 0.001).

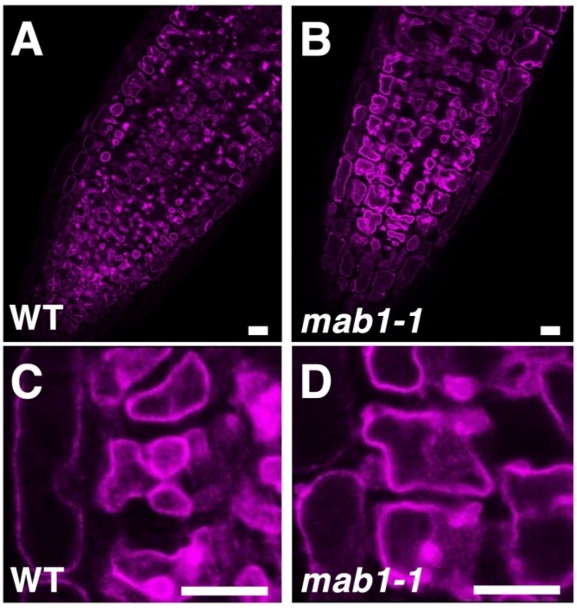

Supplementary Figure S2. ***mab1-1*** **did not affect vacuole morphology**

(**A**–**D**) TagRFP-VAMP713 localization in wild-type (WT) (**A**, **C**) and *mab1-1* (**B**, **D**) root tips of 5-day-old seedlings harboring *VAMP713p:TagRFP-VAMP713*. Scale bars = 10 µm.

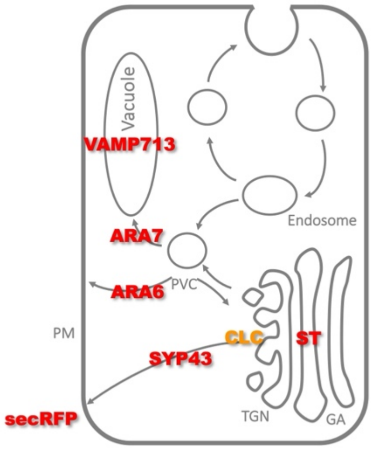

Protein	Gene	Localization	References
ST-mRFP	35S:ST-mRFP	Golgi	Latijnhouwers M., et al., Plant J., 44, 459-470 (2005)
mRFP-SYP43	*SYP43p:mRFP-gSYP43*	TGN & PM	Ebine K., et al., Plant Cell, 20, 3006-3021 (2008)
CLC-mKO	*CLCp:CLC-mKO*	TGN (PM)	Ito E., et al., Plant J., 69, 204-216 (2012)
ARA6-mRFP	*ARA6p:gARA6-mRFP*	Late Endosome	Ebine K., et al., Nat. Cell Biol., 13, 853-859 (2011)
mRFP-ARA7	*ARA7p:mRFP-gARA7*	Late Endosome	Ebine K., et al., Nat. Cell Biol., 13, 853-859 (2011)
secRFP	*35S:secRFP*	Apoplast	Zheng et al., Plant Cell, 17, 2020-2036 (2005)
TagRFP-VAMP713	*VAMP713p:TagRFP-gVAMP713*	Vacuole	Takemoto et al., PNAS, 115, 2457-2466 (2018)

Supplementary Figure S3. The information about organelle marker lines

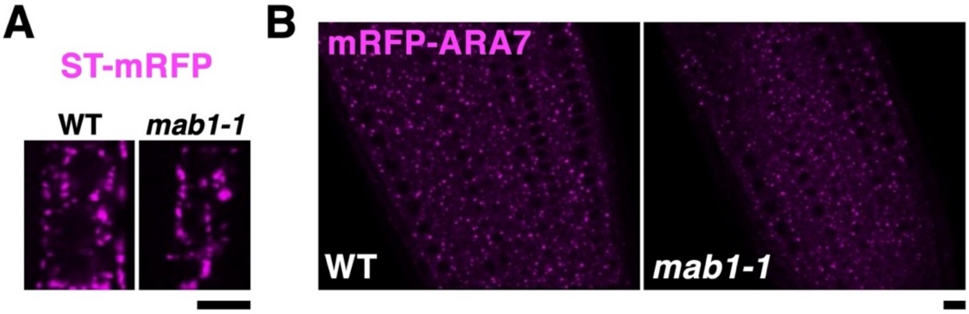

Supplementary Figure S4. The localization of ST-mRFP and mRFP-ARA7 in *mab1-1* roots

(**A**) ST-mRFP localization in epidermal cells of wild-type (WT) (**left**) and *mab1-1* (**right**) root tips of 5-day-old seedlings harboring *35S:ST-mRFP*. (**B**) mRFP-ARA7 localization in wild-type (WT) (**left**) and *mab1-1* (**right**) root tips of 5-day-old seedlings harboring *ARA7p:mRFP-gARA7*. Scale bars = 10 µm.

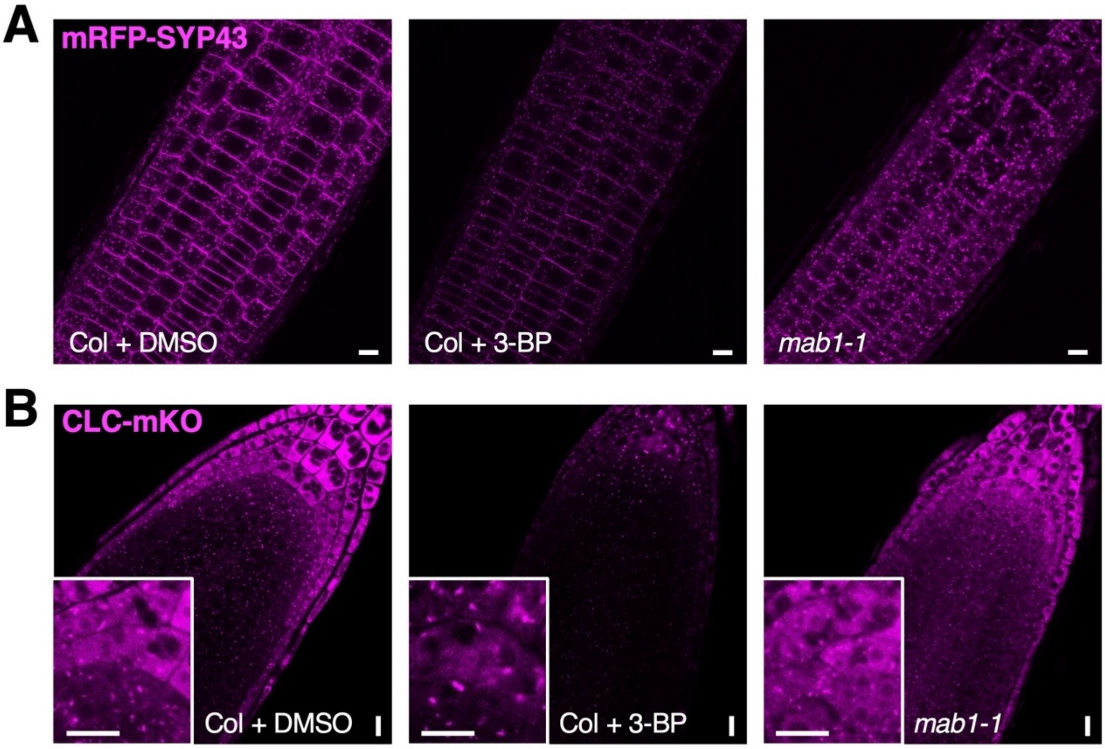

Supplementary Figure S5. The localization of ST-mRFP and mRFP-ARA7 in *mab1-1* roots

(**A**) mRFP-SYP43 localization in epidermal cells of wild-type Col (**left**) and *mab1-1* (**right**) root tips of 5-day-old seedlings harboring *SYP43p:mRFP-gSYP43*. (**B**) CLC-mKO localization in wild-type Col (**left**) and *mab1-1* (**right**) root tips of 5-day-old seedlings harboring *CLCp:CLC-mKO*. Scale bars = 10 µm.

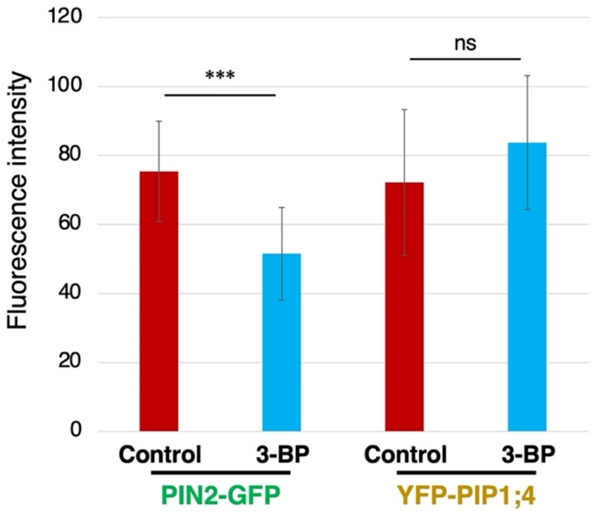

Supplemental Figure S6. 3-BP treatment specifically decreased PIN2-GFP level in the plasma membrane

Comparison of the GFP and YFP signal intensity in the plasma membrane of root epidermis of 5-day-old *PIN2p:PIN2-GFP* and *UBQ10p:YFP-PIP1;4* seedlings treated with ethanol (control) and 3-BP, respectively. Values and bars represent means ± SD of three biological replicates, and differences between the means were assessed for statistical significance using Student's t-test (***P < 0.001, ns means no significant differences).

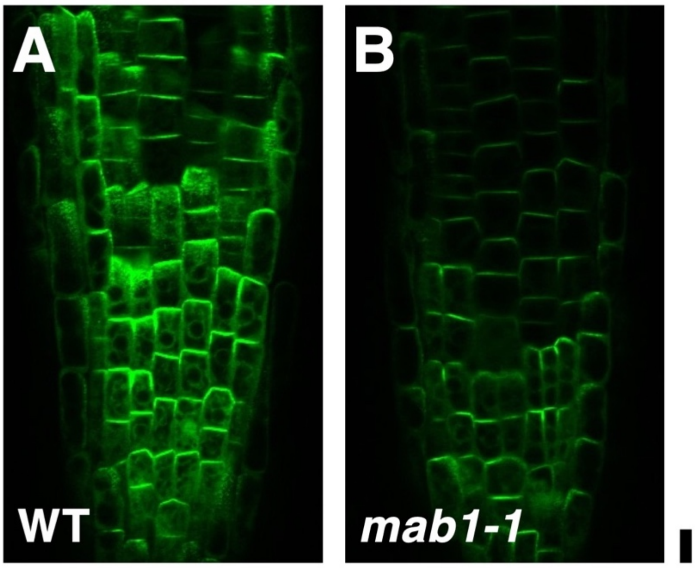

Supplementary Figure S7. Reduced localization of MAB4-GFP in root tips of *mab1-1*

(**A**, **B**) MAB4-GFP localization in wild-type (WT) (**A**) and *mab1-1* (**B**) root tips of 5-day-old seedlings harboring *35S:MAB4-GFP*. Scale bars = 10 µm.

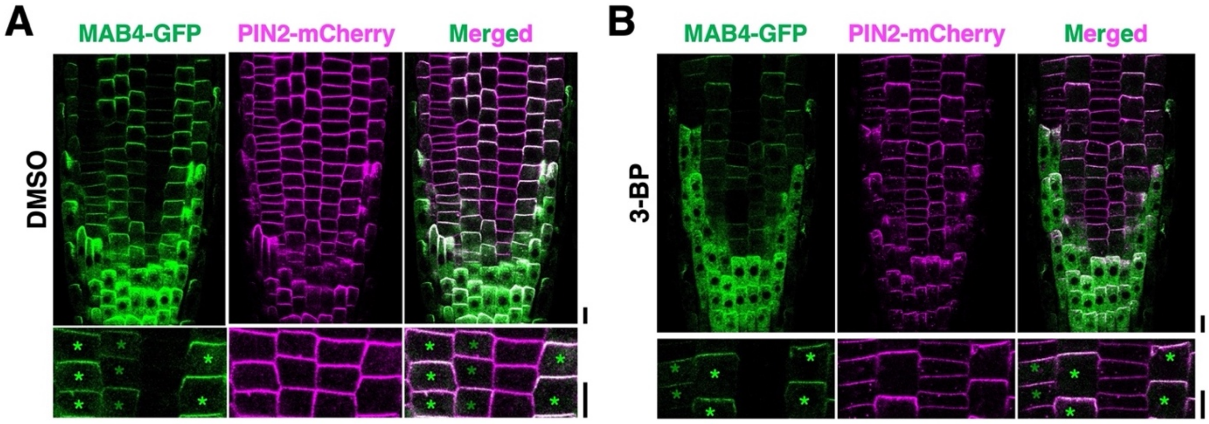

Supplemental Figure S8. Effects of 3-BP treatment on PIN2-mCherry and MAB4-GFP localization

(**A**, **B**) Confocal images of MAB4-GFP (green) and PIN2-mCherry (magenta) in DMSO- (**A**) and 3-BP-treated (**B**) root tips of 5-day-old seedlings harboring *35S:MAB4-GFP* and *PIN2p:PIN2-mCherry*. Merged images of MAB4-GFP and PIN2-mCherry (**right**). Enlarged images of respective figures (**bottom**). Green asterisks indicate cells with MAB4-GFP signals in the plasma membrane. Scale bars = 10 µm.

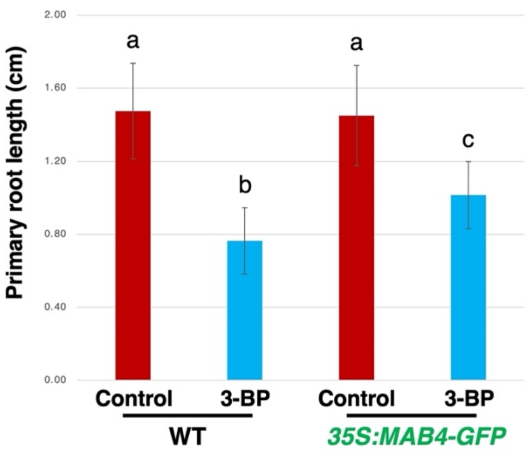

Supplemental Figure S9. Overexpression of *MAB4-GFP* mitigated the effect of 3-BP on root growth in part

Comparison of the root length of *PIN2p:PIN2-mCherry* and *PIN2p:PIN2-mCherry*/*35S:MAB4-GFP* (n≥20) on DMSO- and 0.1 mM 3-BP- containing medium. Error bars represent ± SD. Different letters indicate significant differences at $P < 0.05$ by Tukey-Kramer method.

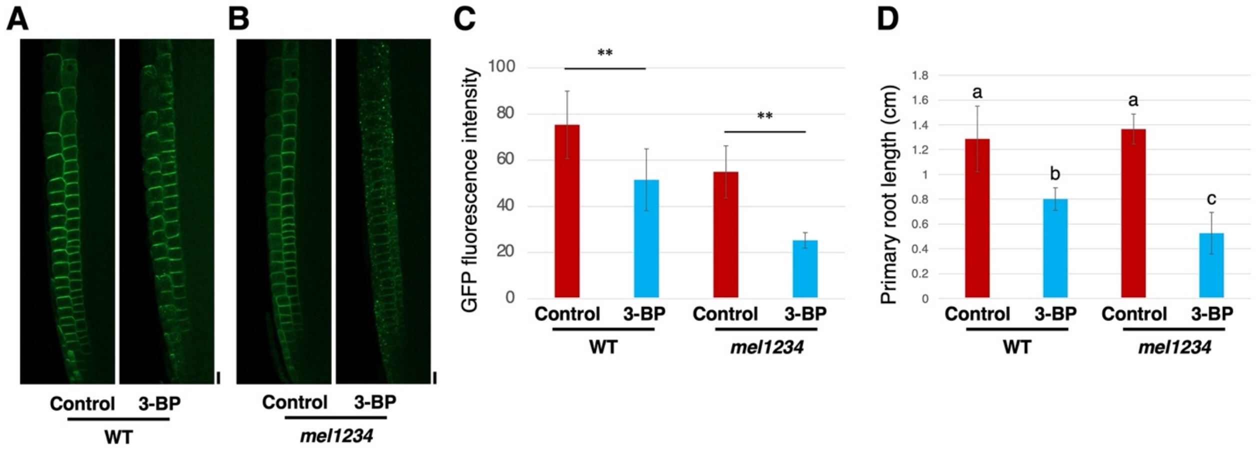

Supplemental Figure S10. Reduced PIN2-GFP localization and root growth in root tips of *mel1 mel2 mel3 mel4*

(**A**, **B**) PIN2-GFP localization in wild-type (WT) (**A**) and *mel1-1 mel2-1 mel3-1 mel4-1* (*mel1234*) (**B**) root tips in 5-day-old seedlings harboring *PIN2p:PIN2-GFP*, treated with ethanol (control) and 3-BP. Scale bars = 10 µm. (**C**) Comparison of the GFP signal intensity in the plasma membrane of root epidermis of 5-day-old *PIN2p:PIN2-GFP* seedlings treated with ethanol (control) and 3-BP in the WT and *mel1234* mutant background, respectively. Values and bars represent means ± SD of three biological replicates, and differences between the means were assessed for statistical significance using Student's *t*-test (**P < 0.01). (**D**) Primary root length of the 5-day-old WT and *mel1234* treated with 0.1 mM 3-BP. Different letters indicate significant differences at P < 0.05 by Tukey-Kramer method.

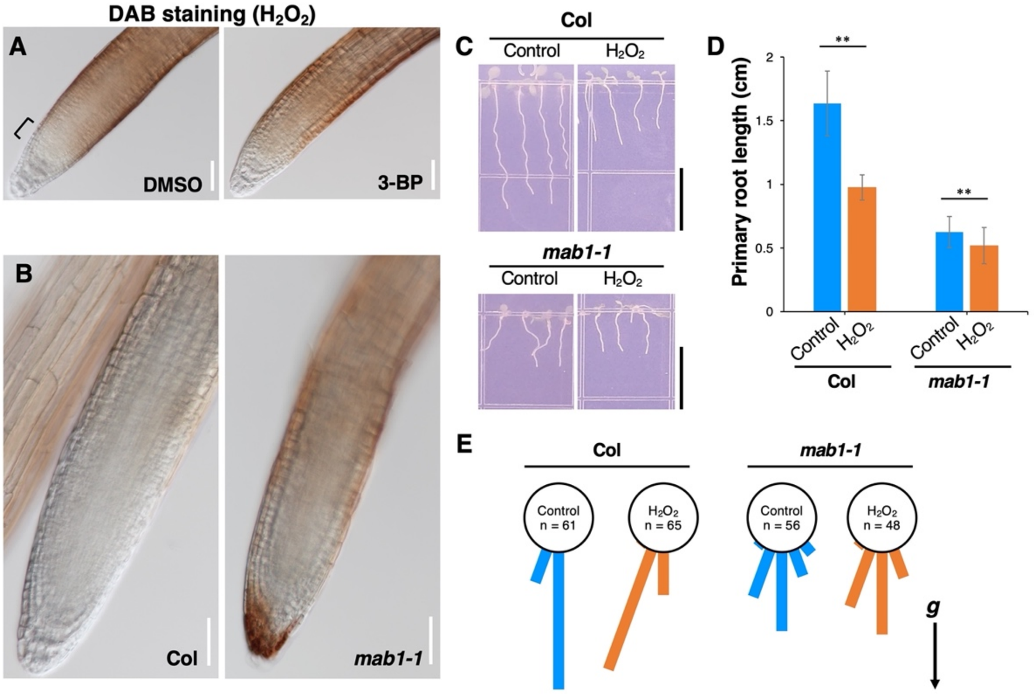

Supplemental Figure S11. TCA cycle impairment increased H_2O_2 level in root tips

(**A**) DAB staining for H2O2 in the primary root of the 5-day-old Col treated with DMSO and 3-BP. (**B**) DAB staining in the primary root of 5-day-old wild-type Col (**left**) and *mab1-1* seedlings (**right**). (**C**) 5-day-old seedlings of Col (**top**) and *mab1-1* (**bottom**) treated with H_2O_2. (**D**) Comparison of primary root length and elongation of 5-day-old seedlings of Col and *mab1-1* treated with H_2O_2. Values and bars represent means ± SD of three biological replicates, and asterisks indicate a significant difference between the means at $P < 0.01$ (two-tailed Student's *t*-test). (**E**) Growth direction of the primary root of 5-day-old seedlings of Col and *mab1-1* on medium containing H_2O_2. The arrow marked with "*g*" represents the direction of gravity. Scale bars = 50 µm in (**A**, **B**), and 1 cm in (**C**).

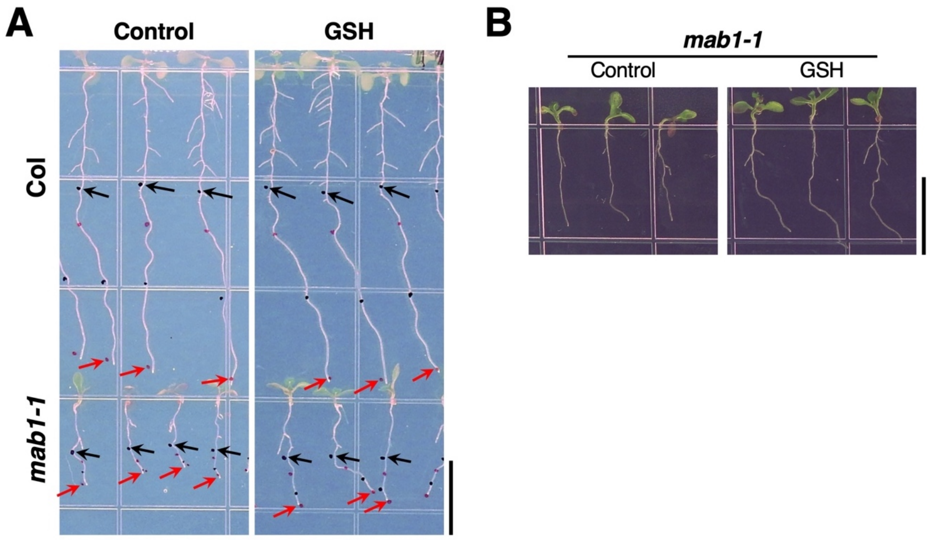

Supplementary Figure S12. Promoted root elongation on GSH-containing medium

(**A**) Seedlings of wild-type Col (**top**) and *mab1-1* (**bottom**) grown on the ½ MS medium containing 1 mM GSH (**right**) 3 days after transfer of 5-day-old seedlings from ½ MS medium. Black arrows show the position of root tips just after transfer, whereas red arrows indicate root tip positions 3 days after transfer. (**B**) 8-day-old seedlings of the *mab1-1* mutant on the ½ MS medium containing 1 mM GSH. Scale bars = 1 cm.

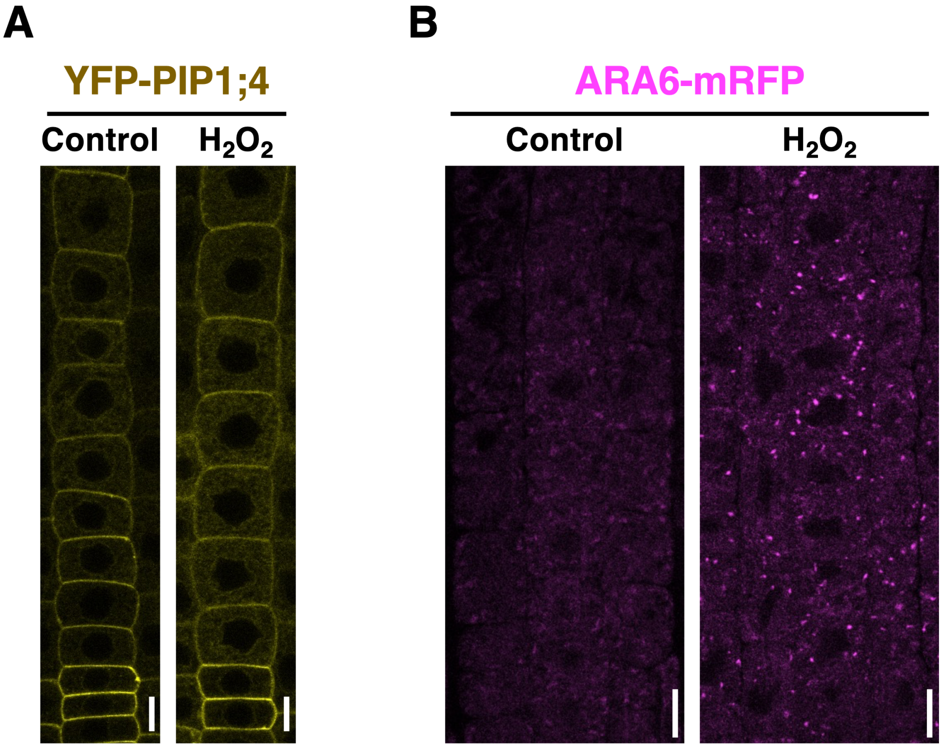

Supplementary Figure S13. H_2O_2 treatment did not affect the localization of YFP-PIP1;4, but ARA6-mRFP, in root epidermis

(A, B) YFP-PIP1;4 (A) and ARA6-mRFP (B) localization in root epidermis of 5-day-old seedlings harboring *UBQ10p:YFP-PIP1;4* and *ARA6p:gAR6-mRFP*, respectively, treated with water (control) and H_2O_2. Scale bars = 10 µm.

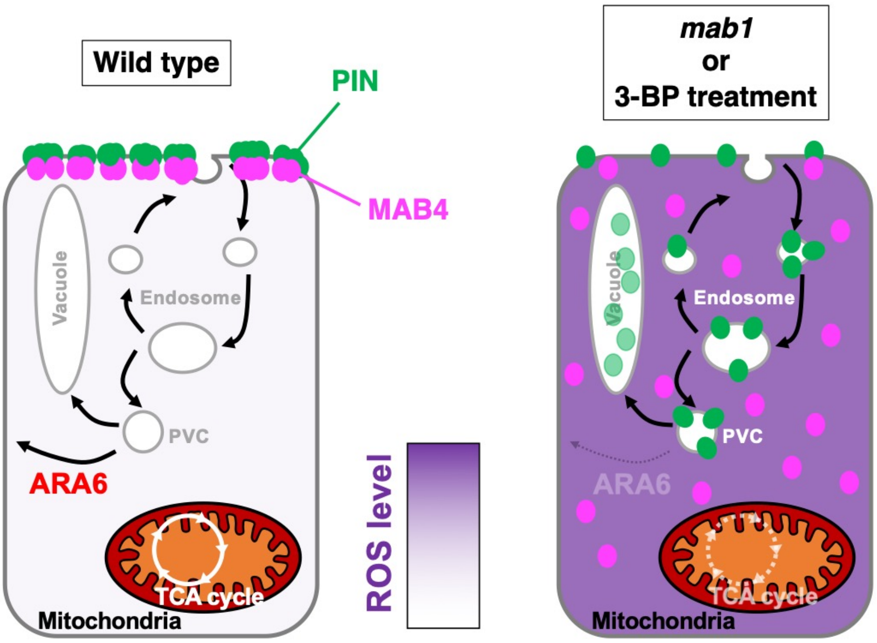

Supplemental Figure S14. Schematic diagrams of PIN trafficking and ROS levels in wild-type, and *mab1* or 3-BP-treated root cells

In wild-type cells, MAB4 blocks PIN internalization in the plasma membrane (left). In cells with impaired TCA cycle, ROS accumulation reduces MAB4-dependent block of PIN internalization and ARA6-dependent endocytic recycling to the plasma membrane, resulting in vacuolar targeting of PIN proteins (right).
